# *Chlamydia trachomatis* Infection, Immunity, and Pregnancy Outcome

**DOI:** 10.1155/S1064744997000203

**Published:** 1997

**Authors:** S. S. Witkin, A. Neuer, P. Giraldo, J. Jeremias, V. Tolbert, I. L. Korneeva, D. Kneissl, A. M. Bongiovanni

**Affiliations:** Department of Obstetrics and Gynecology Cornell University Medical College 515 East 71st Street New York NY 10021 USA

## Abstract

*Chlamydia trachomatis* can ascend from the cervix to the fallopian tubes and survive for long periods of time without causing symptoms. The immune response to infection clears the extracellular organisms but leads to development of a persistent intracellular infection. Repeated cycles of productive infection and persistence eventually induce tubal occlusion and infertility. Persistently infected cells continue to synthesize the chlamydial 60 kD heat shock protein (hsp60). Immunity to conserved regions of hsp60 may result in autoimmunity to human hsp60. Expression of hsp60 by the embryo and decidua during early pregnancy may reactivate hsp60-sensitized lymphocytes, disturb pregnancy-induced immune regulatory mechanisms, and lead to immune rejection of the embryo. Due to this mechanism women with tubal infertility who are sensitized to the human hsp60 may have a decreased probability of successful outcome after undergoing in vitro fertilization and embryo transfer.


Infectious Diseases in Obstetrics and Gynecology 5:128–132 (1997)
© 1997 Wiley-Liss, Inc.

### *Chlamydia trachomatis* Infection, Immunity, and Pregnancy Outcome

S.S. Witkin,* A. Neuer, P. Giraldo, J. Jeremias, V. Tolbert, I.L. Korneeva, D. Kneissl, and A.M. Bongiovanni

*Department of Obstetrics and Gynecology, Cornell University Medical College, New York, NY*

Abstract

**Chlamydia trachomatis can ascend from the cervix to the fallopian tubes and survive for long periods of time without causing symptoms. The immune response to infection clears the extracellular organisms but leads to development of a persistent intracellular infection. Repeated cycles of productive infection and persistence eventually induce tubal occlusion and infertility. Persistently infected cells continue to synthesize the chlamydial 60 kD heat shock protein (hsp60). Immunity to conserved regions of hsp60 may result in autoimmunity to human hsp60. Expression of hsp60 by the embryo and decidua during early pregnancy may reactivate hsp60-sensitized lymphocytes, disturb pregnancy-induced immune regulatory mechanisms, and lead to immune rejection of the embryo. Due to this mechanism women with tubal infertility who are sensitized to the human hsp60 may have a decreased probability of successful outcome after undergoing in vitro fertilization and embryo transfer. Infect. Dis. Obstet. Gynecol. 5:128–132, 1997.** © 1997 Wiley-Liss, Inc.

Key words

*Chlamydia trachomatis* infection; immunity; pregnancy outcome

Due to recent advances in assisted reproduction technology many conditions previously considered to eliminate any chance for fertility are now eminently treatable. In vitro fertilization (IVF) bypasses the requirement for patent fallopian tubes and has rapidly become the treatment of choice for women with occluded or damaged fallopian tubes. However, it is only beginning to be appreciated that the cause of the tubal blockage may also negatively influence postfertilization events. In this article, we focus on the major cause of tubal occlusion in the United States and western Europe, *Chlamydia trachomatis* infections. We delineate the unique aspects of intracellular chlamydial persistence, the immune-mediated mechanism responsible for *C. trachomatis*-induced tubal damage and, lastly, the negative consequences these immune events may have for subsequent fertility after IVF and embryo transfer.

### *C. TRACHOMATIS* PERSISTENCE

*C. trachomatis* can exist in two distinct morphological types. The extracellular elementary body is the infectious morphotype, while the intracellular reticulate body is the replicating form of the organism. *C. trachomatis* can only divide and reproduce within mammalian cells and is therefore classified as an obligate intracellular microorganism. This ability to reside within mammalian cells allows *C. trachomatis* to evade detection by the host's immune system. Furthermore, the organism has developed mechanisms to persist under adverse conditions. Untreated *C. trachomatis* infections have been shown to persist asymptomatically in the fe-

Contract grant sponsor: R.K. Mellon Family Foundation.
*Correspondence to: Dr. Steven S. Witkin, Department of Obstetrics and Gynecology, Cornell University Medical College, 515 East 71st Street, New York, NY 10021.

Received 1 October 1997

Accepted 21 October 1997

male genital tract for years,[1,2] and this organism has been isolated from fallopian tubes of infertile women with tubal obstruction who had no history of a symptomatic pelvic infection.[3]

There is also substantial evidence that *C. trachomatis* can persist in vivo in an intracellular form that cannot be propagated by in vitro culture.[4,5] *C. trachomatis* DNA and protein have been identified in culture-negative fallopian tubes from women with postinfectious tubal infertility.[6] The mechanism underlying chlamydial persistence in an unculturable form has been elucidated in in vitro systems; evidence for a similar mechanism existing in vivo is still lacking. Exposure of chlamydial-infected cells to tumor necrosis factor alpha (TNF-α),[7] interferon gamma (IFN-γ),[8] or some antibiotics[5] leads to development of large aberrant reticulate bodies which are incapable of replication. These forms remain viable, however, and when the cytokine or antibiotic is removed the aberrant forms convert back to typical reticulate bodies and the chlamydial life cycle resumes. With each repeated cycle of productive infection and persistence the tubal epithelium becomes increasingly damaged due to cell destruction and the elaboration of pro-inflammatory mediators. Eventually, scarring becomes sufficiently pronounced to occlude the tube. This scheme of tubal damage is outlined in Table 1.

**TABLE 1. Proposed mechanism of *C. trachomatis*-induced fallopian tube occlusion**

| |
|---|
| Asymptomatic *C. trachomatis* infection ascends to the fallopian tubes. |
| Extracellular chlamydial elementary bodies activate the host's immune response, and IFN-γ, TNF-α, and other pro-inflammatory cytokines are released. |
| The extracellular infection is cleared by the immune system. IFN-γ and TNF-α suspend intracellular chlamydial reticulate body development but do not affect viability. TNF-α and other inflammatory mediators locally damage the tubal epithelia. |
| When the extracellular infection is cleared, IFN-γ and TNF-α production ceases and reticulate body development resumes. |
| The reticulate bodies differentiate into elementary bodies, the infected cell is lysed, and other epithelial cells are infected. |
| The elementary bodies again activate the immune response and the resulting cytokine production induces chlamydial persistence and further damage to tubal tissue. |
| Repeated cycles of productive infection and persistence eventually lead to fallopian tube occlusion. |

### *C. TRACHOMATIS* 57 kD HEAT SHOCK PROTEIN AND IMMUNE PATHOGENESIS

In its persistent, unculturable form, *C. trachomatis* synthesizes very little, if any, of its major structural components. However, one chlamydial protein, of 57 kD, continues to be produced and at a rate even higher than under favorable growth conditions.[9] This protein has been shown to be a member of the 60 kD heat shock protein (hsp60) family[10] with extensive amino acid sequence homology to the hsp60s of other bacteria and man.[11] Hsp60 is a highly conserved protein that functions in the intracellular transport and assembly of other proteins. Under conditions of stress or rapid growth its synthesis is greatly enhanced to prevent protein denaturation and incorrect folding and, thereby, maximize the cell's ability to survive.[12]

The chlamydial hsp60 is a potent inducer of inflammation. In guinea pigs and monkeys previously sensitized to *Chlamydia*, introduction of purified chlamydial hsp60 induced a delayed hypersensitivity response.[13,14] This suggested that an immune response to hsp60 might be involved in chlamydial disease pathogenesis. In man, several investigations have established associations between immunity to *C. trachomatis* hsp60 and recurrent salpingitis, tubal occlusion, and ectopic pregnancy.[15–18] Very recent studies have further demonstrated that the presence of antibody to the chlamydial hsp60 was a risk factor for subsequent development of fallopian tube inflammation.[19]

The extensive amino acid sequence homology between the chlamydial and human hsp60 implies that under certain conditions a *C. trachomatis* infection might induce autoimmunity to one's own hsp60. Cell-mediated immunity to synthetic peptides corresponding to regions of the chlamydial hsp60 that were also present in human hsp60 was not observed in women with a recent chlamydial cervicitis and only rarely in women with an initial episode of salpingitis. It was a frequent finding, however, in women with recurrent chlamydial salpingitis.[20] This suggested that repeated or chronic exposure to the chlamydial hsp60 was required for development of specific immunity to hsp60 epitopes shared between *C. trachomatis* and man. Evidence of humoral immunity to conserved hsp60 epitopes has been obtained for women with *C. trachomatis*-associated ecopic pregnancy[21] and infertile women undergoing IVF.[22]

### IMMUNITY TO *C. TRACHOMATIS*, hsp60, AND PREGNANCY OUTCOME

The relationship between prior immunity to *C. trachomatis* and pregnancy outcome remains controversial. We,[23] and others,[24] have presented data consistent with increasing susceptibility to subsequent spontaneous abortion after a chlamydial infection. Circulating anti-chlamydial IgG has also been associated with IVF failures[23,25,26] in some studies but not others.[27] These studies were all performed prior to the knowledge that immunity to the chlamydial hsp60 induces pro-inflammatory responses. A recent review on the possible influence of immunity to hsp60 and infertility has been published.[28]

Whether prior immunity to the chlamydial hsp60 and to hsp60 epitopes also expressed in human hsp60 affects pregnancy outcome has become a critically important question since many women with tubal factor infertility now seek to become pregnant via IVF. A negative influence of hsp60-mediated immunity on pregnancy is a realistic possibility since the embryo[29] and epithelial cells in the maternal decidua[30] both express hsp60 during the early stages of pregnancy. In addition, a murine hybridoma specific for hsp60 was shown to also react with the surface of human trophoblast,[31] suggesting hsp60 expression by these cells.

In a recent study of 198 women undergoing IVF and embryo transfer, cervical IgA antibodies to the *C. trachomatis* hsp60 were identified in 41 (20.7%) patients.[32] The occurrence of this antibody was strongly correlated with unsuccessful IVF outcome. Anti-hsp60 IgA was present in 26.3% of women who did not become pregnant after embryo transfer, 33.3% of women with only transient biochemical pregnancies, 30% of women with spontaneous abortions, and only 7.3% of women with live births. There was no relation between hsp60 antibody status and numbers of oocytes retrieved or fertilized. In a subsequent analysis of these same patients utilizing a synthetic peptide to a region of the chlamydial hsp60 that is also expressed in man (amino acids 260–271), it was demonstrated that the presence of IgA antibodies to this peptide also correlated with IVF failure and was associated with only biochemical pregnancies after embryo transfer.[22]

TABLE 2. Proposed mechanism of hsp60 immunity-related pregnancy loss

| |
|---|
| A persistent chlamydial fallopian tube infection sensitizes the woman to regions of hsp60 present in both *C. trachomatis* and man. |
| During the pre- and peri-implantation stages of pregnancy, host hsp60 is expressed by the embryo and maternal decidua. |
| Host hsp60 expression reactivates lymphocytes previously sensitized to the chlamydial hsp60. |
| The activated lymphocytes release pro-inflammatory cytokines which induce other lymphoid cells to release inflammatory and cytotoxic mediators. |
| Immune system activation disturbs immune regulatory mechanisms necessary to prevent recognition of paternal antigens expressed by the semi-allogeneic embryo. |
| Female immune system recognition of paternal antigens induces rejection of the embryo. |

An analysis of cervical IgA antibodies to the human hsp60 in 91 women seen for an infertility evaluation who were not in an IVF program revealed that 14.3% were antibody positive. Humoral immunity to human hsp60 was associated with a pro-inflammatory immune response at that site. The presence of this antibody correlated with detection of IFN-$\gamma$ and TNF-$\alpha$ in the cervix.[22]

A third study measuring systemic circulating IgG antibody to human hsp60 in infertile women, women with a history of recurrent abortions, and women with primary infertility demonstrated an association between this antibody and recurrent abortion. IgG anti-human hsp60 was present in 11% of fertile women, 12% of infertile women, and 37% of recurrent aborters.[22]

The relation between serum anti-hsp60 antibodies and recurrent abortion suggests that this antibody may interfere with embryo development. In an in vitro mouse model the addition of monoclonal antibody to mammalian hsp60 severely impeded embryo development.[33] In IVF, embryos are cultured in medium containing maternal serum. Thus, if the woman is immunized to conserved hsp60 epitopes, this may interfere with the in vitro development of her embryos.

A proposed mechanism of hsp60 immunity-mediated postfertilization pregnancy loss is outlined in Table 2. Expression of hsp60 by the embryo and/or maternal cells could reactivate lymphocytes previously sensitized to conserved hsp60 epitopes as a result of an asymptomatic *C. trachomatis* infection. This would result in the elaboration of pro-inflammatory cytokines and induction of a localized delayed hypersensitivity response. Interference with immunoregulatory mechanisms necessary for maintenance of the semi-allogeneic embryo would then lead to immune rejection of the embryo. Another possibility is that a persistent, inapparent, intracellular *C. trachomatis* infection is still present in some women and becomes reactivated as a result of pregnancy-induced immune alterations. The host's response to infection would then facilitate immune rejection.

### FUTURE PROSPECTS

The recently recognized negative effects of an asymptomatic female upper genital tract chlamydial infection on pregnancy, the capacity to develop a persistent infection, and the induction of immune sensitization to conserved regions of hsp60 highlight even more the necessity for comprehensive *C. trachomatis* screening of sexually active women. To preserve fertility, this infection must be detected and effectively treated prior to establishment of persistence in the fallopian tubes. Utilization of DNA amplification technology, such as polymerase chain reaction (PCR)-based assays, should also be mandatory since their sensitivity exceeds that of any other detection method. Recent advances, such as the demonstration that specimens obtained from the entrance to the vagina (introitus) were as sensitive as endocervical specimens for *C. trachomatis* detection if PCR was utilized,[34] should greatly expand the numbers of women who can be tested for this organism.

Physicians and other health care professionals must become advocates for state-of-the-art preventative and diagnostic modalities for women and not capitulate to non-professionals interested more in finances than the quality of human lives.

### ACKNOWLEDGMENTS

These studies were supported in part by the R.K. Mellon Family Foundation.

### REFERENCES

1. McCormack WM, Alpert S, McComb DE, Nichols RL, Semine DZ, Zinner SH: Fifteen-month follow-up study of women infected with *Chlamydia trachomatis*. N Engl J Med 300:123–125, 1979.
2. Ruijs GJ, Kauer FM, Jager S, Schroder FP, Schirm J, Kremer J: Epidemiological aspects of chlamydial infections and tubal abnormalities in infertile couples. Eur J Obstet Gynecol Reprod Biol 36:107–116, 1990.
3. Henry-Suchet J, Catalan F, Loffredo V, et al.: Microbiology of specimens obtained by laparoscopy from controls and from patients with pelvic inflammatory disease or infertility with tubal obstruction: *Chlamydia trachomatis* and *Ureaplasma urealyticum*. Am J Obstet Gynecol 138:1022–1025, 1980.
4. Schachter J, Moncada J, Dawson CR, et al.: Nonculture methods for diagnosing chlamydial infection in patients with trachoma: A clue to the pathogenesis of the disease? J Infect Dis 158:1347–1352, 1988.
5. Beatty WL, Byrne GI, Morrison RP: Repeated and persistent infection with *Chlamydia* and the development of chronic inflammation and disease. Trends Microbiol 2: 94–98, 1994.
6. Patton DL, Askienazy-Elbhar M, Henry-Suchet J, et al.: Detection of *Chlamydia trachomatis* in fallopian tube tissue in women with postinfectious tubal infertility. Am J Obstet Gynecol 171:95–101, 1994.
7. Shemer-Avni Y, Wallach D, Sarov I: Inhibition of *Chlamydia trachomatis* growth by recombinant tumor necrosis factor. Infect Immun 56:2503–2506, 1988.
8. Beatty WL, Byrne GI, Morrison RP: Morphologic and antigenic characterization of interferon γ-mediated persistent *Chlamydia trachomatis* infection in vitro. Proc Natl Acad Sci USA 90:3998–4002, 1993.
9. Beatty WL, Morrison RP, Byrne GI: Immunoelectron-microscopic quantitation of different levels of chlamydial proteins in a cell culture model of persistent *Chlamydia trachomatis* infection. Infect Immun 62:4059–4062, 1994.
10. Morrison RP, Belland RJ, Lyng K, Caldwell HD: Chlamydial disease pathogenesis. The 57-kD chlamydial hypersensitivity antigen is a stress response protein. J Exp Med 170:1271–1283, 1989.
11. Morrison RP, Manning DS, Caldwell HD: Immunology of *Chlamydia trachomatis* infections. Immunoprotective and immunopathogenetic responses. In Quinn TC (ed): Sexually Transmitted Diseases. New York: Raven Press, pp 57–84, 1992.
12. Ellis RJ: Stress proteins as molecular chaperones. In van Eden W, Young DB (eds): Stress Proteins in Medicine. New York: Marcel Dekker, pp 1–26, 1996.
13. Morrison RP, Lyng K, Caldwell HD: Chlamydial disease pathogenesis. Ocular hypersensitivity elicited by a genus-specific 57-kD protein. J Exp Med 169:663–675, 1989.
14. Patton DL, Sweeney YT, Kuo CC: Demonstration of delayed hypersensitivity in *Chlamydia trachomatis* salpingitis in monkeys: A pathogenic mechanism of tubal damage. J Infect Dis 169:680–683, 1994.
15. Wager EA, Schachter J, Bavoil P, Stephens RS: Differential human serologic response to two 60,000 molecular weight *Chlamydia trachomatis* antigens. J Infect Dis 162: 922–927, 1990.
16. Brunham RC, Peeling R, Maclean I, Kosseim ML, Paraskevas M: *Chlamydia trachomatis*-associated ectopic pregnancy: Serologic and histologic correlates. J Infect Dis 165:1076–1081, 1992.
17. Witkin SS, Jeremias J, Toth M, Ledger WJ: Cellmediated immune response to the recombinant 57-kDa heat shock protein of *Chlamydia trachomatis* in women with salpingitis. J Infect Dis 167:1379–1383, 1993.

18. Toye B, Laferriere C, Claman P, Jessamine P, Peeling R: Association between antibody to the chlamydial heat-shock protein and tubal infertility. J Infect Dis 168: 1236–1240, 1993.
19. Peeling RW, Kimani J, Plummer F, et al.: Antibody to chlamydial hsp60 predicts an increased risk for chlamydial pelvic inflammatory disease. J Infect Dis 175: 1153–1158, 1997.
20. Witkin SS, Jeremias J, Toth M, Ledger WJ: Proliferative response to conserved epitopes of the *Chlamydia trachomatis* and human 60-kilodalton heat-shock proteins by lymphocytes from women with salpingitis. Am J Obstet Gynecol 171:455–460, 1994.
21. Yi Y, Zhong G, Brunham RC: Continuous B cell epitopes in *Chlamydia trachomatis* heat shock protein 60. Infect Immun 61:1117–1120, 1993.
22. Witkin SS, Jeremias J, Neuer A, et al.: Immune recognition of the 60 kD heat shock protein: Implications for subsequent fertility. Infect Dis Obstet Gynecol 4:152–158, 1996.
23. Licciardi F, Grifo JA, Rosenwaks Z, Witkin SS: Relation between antibodies to *Chlamydia trachomatis* and spontaneous abortion following in vitro fertilization. J Assist Reprod Genet 9:207–210, 1992.
24. Quinn P, Petric M, Barkin M: The prevalence of antibody to *Chlamydia trachomatis* in spontaneous abortion and infertility. Am J Obstet Gynecol 156:291–296, 1987.
25. Rowland G, Forsey T, Moss T, Steptoe P, Hewitt J, Darougar S: Failure of in vitro fertilization and embryo replacement following infection with *Chlamydia trachomatis*. J In Vitro Fertil Embryo Transfer 2:151–155, 1985.
26. Lunenfeld E, Shapiro BS, Sarov B, Sarov I, Insler V, DeCherney A: The association between *Chlamydia*-specific IgG and IgM antibodies and pregnancy outcome in an in vitro fertilization program. J In Vitro Fertil Embryo Transfer 6:222–227, 1989.
27. Torode HN, Wheeler PA, Saunders DM, McPetric RA, Medcalf SC, Ackerman VP: The role of *Chlamydia* antibodies in an in vitro fertilization program. Fertil Steril 48:987–990, 1987.
28. Witkin SS: Stress proteins and infertility. In van Eden W, Young DB (eds): Stress Proteins in Medicine. New York: Marcel Dekker, pp 301–306, 1996.
29. Bensuade O, Morange M: Spontaneous high expression of heat-shock proteins in mouse embryonal cells and ectoderm from day 8 mouse embryos. EMBO J 2:173–177, 1983.
30. Mincheva-Nilsson L, Baranov V, Yeung MM, Hammarstrom S, Hammarstrom ML: Immunomorphologic studies of human decidua-associated lymphoid cells in normal early pregnancy. J Immunol 152:2020–2032, 1994.
31. Heybourne K, Fu YX, Nelson A, Farr A, O'Brien R, Born W: Recognition of trophoblasts by $\gamma\delta$ T cells. J Immunol 153:2918–2926, 1994.
32. Witkin SS, Sultan KM, Neal GS, Jeremias J, Grifo JA, Rosenwaks Z: Unsuspected *Chlamydia trachomatis* infection and in vitro fertilization outcome. Am J Obstet Gynecol 171:1208–1214, 1994.
33. Neuer A, Spandorfer S, Giraldo P, et al.: Immune sensitization to the 60 kD heat shock protein and pregnancy outcome. Infect Dis Obstet Gynecol 5:155–158, 1997.
34. Witkin SS, Inglis SR, Polaneczky M: Detection of *Chlamydia trachomatis* and *Trichomonas vaginalis* by polymerase chain reaction in introital specimens from pregnant women. Am J Obstet Gynecol 175:165–167, 1996.
